# Generalizability of PGS_313_ for breast cancer risk in a Los Angeles biobank

**DOI:** 10.1016/j.xhgg.2024.100302

**Published:** 2024-05-03

**Authors:** Helen Shang, Yi Ding, Vidhya Venkateswaran, Kristin Boulier, Nikhita Kathuria-Prakash, Parisa Boodaghi Malidarreh, Jacob M. Luber, Bogdan Pasaniuc

**Affiliations:** 1 Division of Internal Medicine, Ronald Reagan UCLA Medical Center, Los Angeles, CA, USA; 2 Department of Computer Science and Engineering, University of Texas at Arlington, Arlington, TX, USA; 3 Bioinformatics Interdepartmental Program, University of California, Los Angeles, Los Angeles, CA, USA; 4 Department of Oral Biology, UCLA School of Dentistry, Los Angeles, CA, USA; 5 Division of Cardiology, Department of Medicine, Ronald Reagan UCLA Medical Center, Los Angeles, CA, USA; 6 Division of Hematology-Oncology, Department of Medicine, Ronald Reagan UCLA Medical Center, Los Angeles, CA, USA; 7 Department of Computer Science and Engineering, University of Texas at Arlington, Arlington, TX, USA; 8 Multi-Interprofessional Center for Health Informatics, University of Texas at Arlington, Arlington, TX, USA; 9 Department of Bioengineering, University of Texas at Arlington, Arlington, TX, USA; 10 Bioinformatics Interdepartmental Program, University of California, Los Angeles, Los Angeles, CA, USA; 11 Department of Pathology and Laboratory Medicine, David Geffen School of Medicine, University of California, Los Angeles, Los Angeles, CA, USA; 12 Department of Human Genetics, David Geffen School of Medicine, University of California, Los Angeles, Los Angeles, CA, USA; 13 Department of Computational Medicine, David Geffen School of Medicine, University of California, Los Angeles, Los Angeles, CA, USA; 14 Institute of Precision Health, University of California, Los Angeles, Los Angeles, CA, USA

**Keywords:** Polygenic scores, bioinformatics, breast cancer, cancer risk prediction, PRS313, genetic admixture, biobank, big data

## Abstract

Polygenic scores (PGSs) summarize the combined effect of common risk variants and are associated with breast cancer risk in patients without identifiable monogenic risk factors. One of the most well-validated PGSs in breast cancer to date is PGS_313_, which was developed from a Northern European biobank but has shown attenuated performance in non-European ancestries. We further investigate the generalizability of the PGS_313_ for American women of European (EA), African (AFR), Asian (EAA), and Latinx (HL) ancestry within one institution with a singular electronic health record (EHR) system, genotyping platform, and quality control process. We found that the PGS_313_ achieved overlapping areas under the receiver operator characteristic (ROC) curve (AUCs) in females of HL (AUC = 0.68, 95% confidence interval [CI] = 0.65–0.71) and EA ancestry (AUC = 0.70, 95% CI = 0.69–0.71) but lower AUCs for the AFR and EAA populations (AFR: AUC = 0.61, 95% CI = 0.56–0.65; EAA: AUC = 0.64, 95% CI = 0.60–0.680). While PGS_313_ is associated with hormone-receptor-positive (HR+) disease in EA Americans (odds ratio [OR] = 1.42, 95% CI = 1.16–1.64), this association is lost in African, Latinx, and Asian Americans. In summary, we found that PGS_313_ was significantly associated with breast cancer but with attenuated accuracy in women of AFR and EAA descent within a singular health system in Los Angeles. Our work further highlights the need for additional validation in diverse cohorts prior to the clinical implementation of PGSs.

## Teaser

We investigated the generalizability of PGS_313_ in predicting the presence of breast cancer in American women of European, African, Asian, and Latinx ancestry within one institution. PGS_313_ had attenuated accuracy in African and Asian females, highlighting the need for validation in diverse cohorts prior to the clinical implementation of polygenic scores.

## Introduction

The US Preventive Services Task Force recommends that breast cancer screening start at 50 years old, based on studies showing that 90% of breast cancer cases are diagnosed after this age.^1^ Unfortunately, this also means that 10% of cases will be missed per conventional guidelines, equating to approximately 10,000 missed cases in the US annually.^2^ As such, researchers have been working to develop new methods of identifying patients at risk of developing early-onset breast cancer. One well-known approach is the Gail model, which uses clinical, family history, and demographic information to calculate individual breast cancer risk but suffers from poor accuracy; in a meta-analysis across 26 studies and 29 datasets, a modified version of the Gail model had areas under the receiver operator characteristic (ROC) curve (AUCs) for American, Asian, and European females of 0.61 (95% confidence interval [CI] = 0.59–0.63), 0.55 (95% CI = 0.52–0.58), and 0.58 (95% CI = 0.55–0.62), respectively.^3^^,^^4^

Another avenue has been identifying carriers of genes such as *BRCA1*, *BRCA2*, *PTEN*, *TP53*, *CDH1*, and *STK11*, which are associated with a 2- to 3-fold elevated risk in developing breast cancer over a patient’s lifetime.^5^ However, the vast majority (>75%) of patients with breast cancer do not have any identifiable monogenic risk factors and thus will not benefit from this approach. Over the past decade, polygenic scores (PGSs) were introduced as a potential solution by summarizing the combined effect of multiple common risk variants that have been individually associated with small yet elevated breast cancer risk.^6^ Several PGSs have been developed to predict and stratify breast cancer risk, including a recent paper showing that at the top 5th percentile of one PGS had a genetic risk of similar magnitude to some monogenic etiologies.^7^ Prior work has estimated that the theoretically best PGS, if the effect sizes of all common SNPs were known with certainty, would explain ∼41% of the familial risk of breast cancer.^8^

One of the most validated PGSs in breast cancer to date is the PGS_313_ by Mavaddat et al., which was developed from a Northern European biobank (*N* = 33,673 cases and *N* = 33,381 controls). When incorporating family history and age of diagnosis, the PGS_313_ achieved an odds ratio (OR) of 1.61 (95% CI = 1.57–1.65) and an AUC of 0.63 (95% CI = 0.63–0.65).^9^ Subsequent work has demonstrated an attenuated effect of PGS_313_ in African females; for example, Cong et al. found that for 33,594 women of European ancestry (EA) and 2,801 women of African (AFR) ancestry (AA) across 9 institutions, the PGS_313_ alone achieved a higher AUC for European females (0.60, 95% CI = 0.59–0.61) than for AFR females (0.55, 95% CI = 0.51–0.58).^10^^,^^11^

In this paper, we aim to further investigate the generalizability of the PGS_313_ for American women of AFR, Asian, and Latinx ancestry within one institution, leveraging a singular electronic health record (EHR) system, genotyping platform, and quality control process.

## Results

Our study included 18,627 women, including 1,156 with AA, 11,873 women with EA, 2,010 with East Asian ancestry (EAA), and 3,303 with Hispanic ancestry (HL), as presented in Table 1. The majority of cases were identified as hormone-receptor positive (HR+) (AA: 71%; EA: 72%; EAA: 65%; HL: 65%), and the prevalence across ancestries was comparable to the latest SEER registry showing that 70% of breast cancer subtypes are HR+.^12^ Similarly, we found prevalences of human epidermal growth factor receptor 2-positive (HER2+) disease that were also comparable to SEER registries showing a prevalence of 10.8%. For 1,285 out of 2,080 cases, we did not have any prescription data, as these patients may have undergone cancer treatment outside of the UCLA network.

**Table 1:** Participant characteristics

	AA	EA	EAA	HL
Controls	1,032	10,407	1,803	3,083
Cases (% of total)	124 (10)	1,466 (12)	207 (10)	220 (6.7)
Age at diagnosis, mean (SD)	55 (17)	55 (17)	52 (17)	48 (17)
Cases with prescription data (% of total)	86 (52)	1,241 (58)	188 (64)	166 (58)
HR+ (% of cases with prescription data)	60 (71)	979 (72)	129 (65)	115 (65)
HER2+ (% of cases with prescription data)	11 (12)	130 (10)	26 (14)	18 (11)
OS in days, mean (SD)	2,862 (1,922)	2,741 (1,973)	2,569 (1,427)	2,911 (2,149)

Participants were females drawn from the UCLA ATLAS Biobank (*N* = 18,627), which is linked to UCLA medical records from 2013 to present day. Cases and controls were identified based on ICD-9 and ICD-10 coding corresponding to breast cancer. Age at diagnosis was based on the date at which the ICD code appeared in a patient’s chart, which was then used to calculate the overall survival (OS), with the day of death or present day as an endpoint. Breast cancer subtypes were identified based on prescriptions ordered.

### Association of PGS_313_ with breast cancer risk in various ancestries

**Figure 1:**
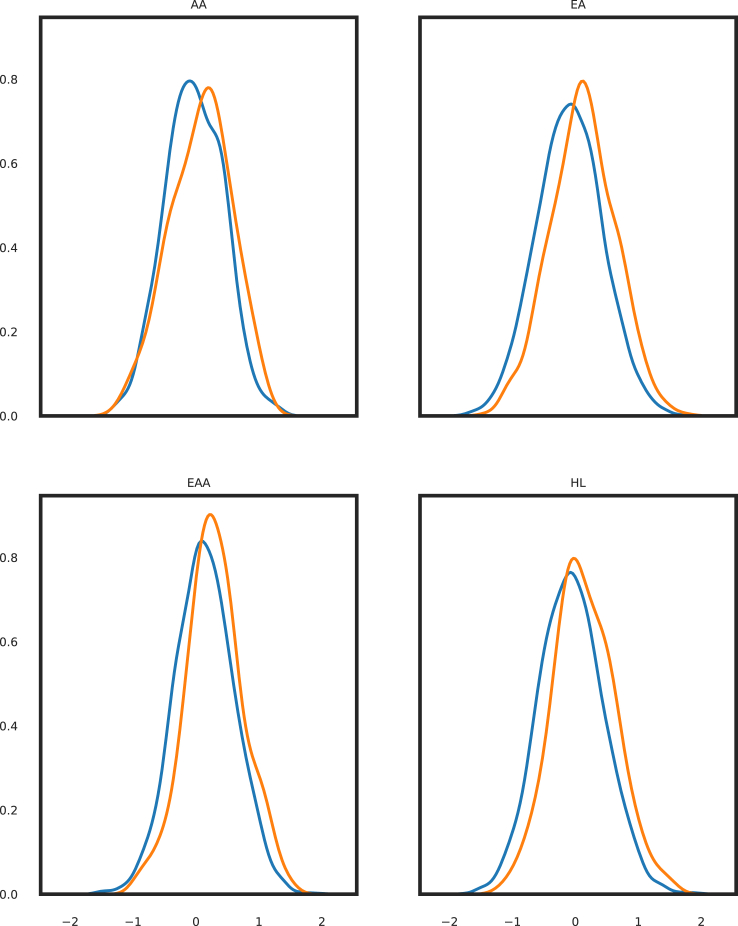
Distribution of PGS_313_ in cases and controls Kernel distribution estimation plots of PGS_313_ scores in cases and controls by genetically inferred ancestry (GIA). The orange curves represent scores for cases and blue curves represent scores for controls. The raw PGS_313_ scores of the European population (European ancestry [EA]) was normalized to a standard deviation of 1 and a mean of 0. The remaining GIAs were normalized to the average and standard deviation of EA samples.

For each genetically inferred ancestry (GIA) group, the PGS followed a normalized distribution with the EAA cohort having a higher mean, as illustrated in Figure 1 (AA: *N* = 1,156, mean = −0.02, SD = 0.46; EA: *N* = 11,873, mean = −0.06, SD = 0.53; EAA: *N* = 2,010, mean = 0.15, SD = 0.47; HL: *N* = 3,303, mean = −0.06, SD = 0.53). We found statistically significant associations of PGS_313_ with overall breast cancer risk across all ancestries. All GIAs had overlapping ORs (AA: OR = 1.31, 95% CI = 1.05–1.64; EA: OR = 1.52, 95% CI = 1.23–1.72; EAA: OR = 1.46, 95% CI = 1.23–1.61; HL: OR = 1.51, 95% CI = 1.31–1.75). These also overlap with Mavaddat et al.’s reported OR in Europeans (OR = 1.61, 95% CI = 1.57 to 1.65).

**Figure 2:**
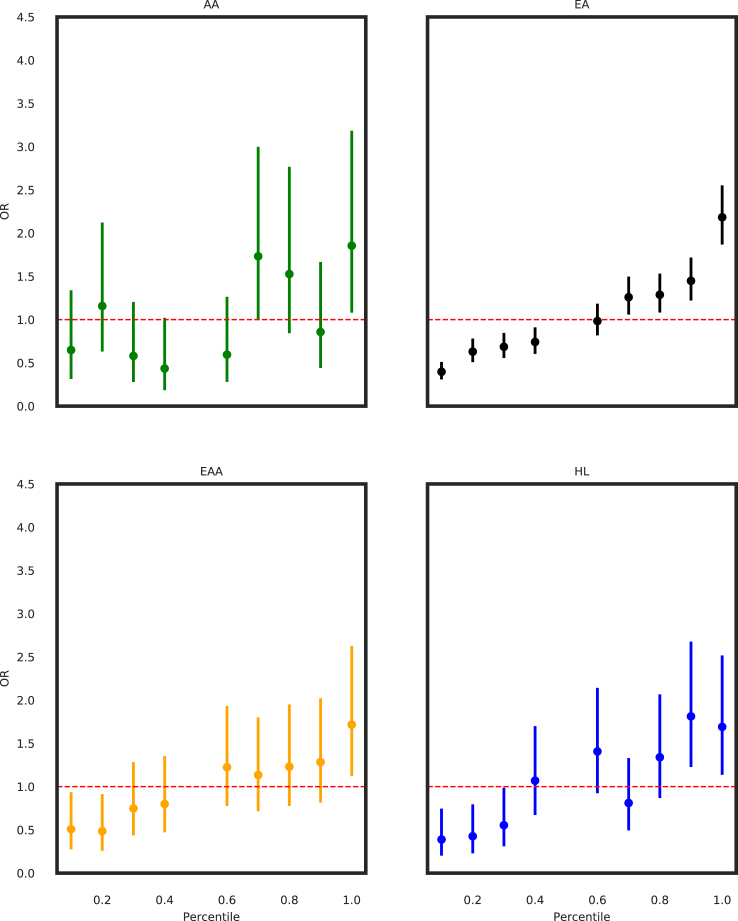
Association of PGS_313_ deciles with breast cancer relative to the 50th percentile Association between PGS_313_ and breast cancer diagnoses in American women of African (AA), European (EA), East Asian American (EAA), and Hispanic (HL) ancestry, based on GIAs, where the OR is plotted on the y axis and percentiles of the PGS_313_ are plotted on the x axis. ORs and 95% confidence intervals are shown. ORs are for different deciles of the PGS relative to the 50th percentile of the PGS.

As seen in Figure 2, for all GIAs, the ORs of the PGS_313_ was largest at the extremes PGS distribution and the 95% CIs overlap among the four GIAs (AA: OR = 1.85, 95% CI = 1.08–3.2; EA: OR = 2.1, 95% CI = 1.9–2.6; EAA: OR = 1.7, 95% CI = 1.1–2.6; HL: OR = 1.7, 95% CI = 1.1–2.5). For the AA population, we found an attenuated effect of the PGS and at the extremes of its distribution (>95th percentile) relative to other GIAs, although this analysis was limited by small sample sizes (data not shown).

To confirm that differences in ORs were not due to sample size imbalances, we conducted an ensemble downsampling experiment. All GIAs were downsampled into 500 batches consisting of 124 cases and 124 controls each, which were randomly selected, as this was the number of cases in the AA cohort, which had the fewest. As shown in Table S4, for each GIA, the batched OR overlapped with the raw OR, suggesting that differences in raw ORs across GIAs are less likely due to differences in sample size.

### Discriminative accuracy of the PGS_313_

The discriminative accuracy of the PGS for any type of breast cancer, as measured by the AUC, was highest in the EA population at 0.70 (95% CI = 0.69–0.71), which is slightly higher than 0.63 (95% CI = 0.63–0.65) as reported by Mavaddat et al. Relative to the EA population, the AUC for the HL population was similar, whereas those for the AFR and EAA populations were lower (AFR: AUC = 0.61, 95% CI = 0.56–0.65; EAA: AUC = 0.64, 95% CI = 0.60–0.680; HL: AUC = 0.68, 95% CI = 0.65–0.71). There was no statistical difference in the AUCs for the EA and HL populations (HL: AUC = 0.68, 95% CI = 0.65–0.71), as determined by their overlapping 95% CIs. In contrast, the AFR and EAA populations were statistically different, as determined by their lower AUCs and non-overlapping 95% CIs, relative to the EA and HL populations (AFR: AUC = 0.61, 95% CI = 0.56–0.65; EAA: AUC = 0.64, 95% CI = 0.60–0.680).

### Association of PGS_313_ with breast cancer subtypes in various ancestries

Recent work in European females has shown that HR+ breast cancer is associated with PGS_313_ and a lower risk genetic signature.^13^ As with prior work, we found that for European patients, the PGS_313_ was associated with HR+ disease; however, it was not associated with HR+ disease in Hispanic, AFR, or Asian American females with breast cancer (Table 2). In contrast, among Hispanic Americans alone, HER2+ disease was instead associated with PGS_313_ with a higher OR than for all breast cancer risk (OR = 2.47, 95% CI = 1.39–4.41), although this analysis was limited, as there were only 18 patients within our cohort that were both Hispanic and HER2+.

**Table 2:** Association of PRS_313_ with breast cancer with HR+ and HER2+ disease by genetically inferred ancestry

	HR−	HR+	OR	Lower CI	Upper CI
AA	15	51	0.71	0.36	1.40
EA	176	768	1.42	1.16	1.64
EAA	22	114	1.38	0.41	1.20
HL	33	106	0.70	0.91	2.20

	HER2−	HER2+	OR	Lower CI	Upper CI
AA	55	11	2.26	0.96	5.33
EAS	814	130	0.95	0.79	1.15
EAA	110	26	1.02	0.62	1.66
HL	121	18	2.47	1.39	4.41

The top section shows observed ORs for the PGS_313_ in predicting hormone-receptor-positive (HR+) or hormone-receptor-negative (HR−) breast cancer across the four GIAs. Logistic regression was performed to predict the labeling of specific subtypes among patients with breast cancer only. The bottom section shows observed ORs for the PGS_313_ in predicting HER+ or HER2− breast cancer across the four GIAs. Columns 2 and 3 reflect positive cases (HR+/HER2+) and negative cases (HR−/HER2−), respectively. PGS313 is only predictive of HR+ disease in European American patients (*p* = 0.0002) but not other ancestries. PGS_313_ is only statistically significant in predicting HER2+/− disease for the Hispanic American cohort, as indicated by the OR’s 95% CI not crossing 1.

### Survival analysis

To confirm that our estimates of overall survival (OS) were reliable, we first confirmed that OS is appropriately associated with whether or not a patient had received chemotherapy, as a hallmark of more aggressive and often metastatic disease (Figure S1). We found that in European patients, the PGS_313_ alone as a predictor fails to stratify patients by survival time by Kaplan-Meier analysis when comparing patients with breast cancer above (*N* = 280) or below (*N* = 651) the 70th percentile with a log-rank *p* value of 0.38 (Figure S2A). We chose to compare above and below the 70th percentile of PGS313 as, starting at this threshold, the OR was noted to be statistically greater than 1 for the EA cohort, as seen in Figure 2. Of note, we found similar results when evaluating different thresholds, such as comparing the top vs. bottom 50th percentiles and the top 90th vs. bottom 10th percentiles. For European patients, after accounting for whether a patient had received chemotherapy, age of diagnosis, HER2+, and PR+ disease using a multivariate Cox proportional hazard model, PGS_313_ was also no longer predictive of OS (Figure S2B). This is consistent with recent work involving a cohort of European (*N* = 98,397) and Asian (*N* = 12,920) females with breast cancer, which found that in European patients, PGS_313_ was no longer associated with OS after adjusting for breast cancer subtype and tumor grade.^13^

## Discussion

In this paper, we investigated the generalizability of the PGS_313_ for American women of AFR, Asian, and Latinx ancestry within one institution. For females of EA, we arrived at overlapping estimates of OR (1.5; 95% CI = 1.2–1.7) when compared to Mavaddat et al. (1.61; 95% CI = 1.57–1.65). Consistent with prior studies, we found that the PGS_313_ was still associated with breast cancer across all ancestries but with an attenuated effect in females of AFR and Asian ancestry; the PGS_313_ achieved equivalent AUCs in females of Latinx ancestry (AUC = 0.68, 95% CI = 0.65–0.71) and EA (AUC = 0.70, 95% CI = 0.69–0.71), with lower AUCs for the AFR and EAA populations (AFR: AUC = 0.61, 95% CI = 0.56–0.65; EAA: AUC = 0.64, 95% CI = 0.60–0.680). This may be due to Latinx individuals having a greater proportion of EA ancestry than Asians and AFR individuals, as we found that the HL cohort had higher levels of EA ancestry relative to all other GIAs (Figure S4).

Consistent with prior work, we found that for European American patients, PGS_313_ is associated with HR+ disease.^9^^,^^13^ For AFR, Hispanic, and Asian American patients, this association is lost, although rates of HR+ disease were lower in these cohorts relative to European Americans. While we unexpectedly found that for Hispanic Americans alone, the PGS_313_ was associated with HER2+ disease, given our cohort’s limited size, further investigation regarding this association is warranted.

Similarly, as with recent work on PGS_313_, we found that it fails to stratify European patients by OS.^14^ One possible explanation is that the impact of PGS_313_ on OS may be confounded by other genetic risk factors, many of which have yet to be identified; several recent papers have found that PGS_313_ stratifies breast cancer risk in *CHEK2*, *PALB2*, and *ATM* carriers but not *BRCA1*/2 carriers.^15^^,^^16^ In other words, the value of PGS_313_ may be in stratifying carriers of low-penetrance risk variants but may fail to stratify those with highly penetrant variants who will go on to develop breast cancer regardless. While we were not able to confirm this in our study given the few number of risk carriers in our cohort, we hope to validate this in future studies.

There are many limitations of this study to consider. Our cohort contained fewer non-European than European samples, and thus analyses at the upper extremes and within subtypes were limited. We also could not estimate the absolute risk of developing breast cancer due to the lack of longitudinal outcomes data. Furthermore, many covariates such as age of diagnoses, cancer subtype, and OS were not available in the medical record as structured data and were thus calculated by proxy methods. Nevertheless, we were able to confirm that these estimates resulted in expected observations, suggesting their reliability, such as the expected prevalence rates among breast cancer subtypes and shorter survival times for patients recieving chemotherapy versus those without due to more aggressive disease.

In summary, we found that PGS_313_ was significantly associated with breast cancer in American females of diverse ancestries but with attenuated accuracy in women of AFR and Asian descent within a singular yet diverse biobank in Los Angeles. While the PGS_313_ is associated with HR+ disease in European Americans, this association is lost in AFR, Hispanic, and Asian Americans. For Hispanic Americans, PGS_313_ may be instead associated with HER2+ disease, although due to small numbers, additional studies will be critical in validating these findings. Our work further highlights the need for additional validation in diverse cohorts prior to clinical implementation of PGSs and the need for new methods that can address differences in genomic admixture.

## Subjects and methods

### Study participants

The participants included in this cohort study were females at birth drawn from the UCLA ATLAS Biobank (*N* = 18,627), which is linked to electronic medical record data and has been described previously.^17^ SNPs were genotyped on a genome-wide array and imputed to the TOPmed reference panel. We identified breast cancer cases and controls using ICD-9 and ICD-10 codes corresponding to Phecode X 105.1, specifying “malignant neoplasm of the breast, female,” which maps ICD codes to clinically meaningful phenotypes.^17^ Patient recruitment and sample collection has been approved by the UCLA Insitutional Review Board (UCLA IRB) IRB#17-001013.

We previously identified five GIAs based on principal-component analysis and k-means clustering including African Americans, Hispanic Latino Americans, East Asian Americans, European Americans, and South Asian Americans (SAA).^18^ The SAA population was not included for further analysis due to case counts being significantly underpowered. As we did not have access to individual-level pathology results, we identified HR+ or HER2+ breast cancer based on prescription data correlating to either subtype.

### PGS models

The PGS was calculated based on an additive model using effect size estimates from the PGS as initially developed by Mavaddat et al., which are available under the entry PGS000004 within The PGS Catalog:^19^

(Equation 1) $$\text{PGS}=\beta_{1}x_{1}+\beta_{2}x_{2}+\ldots \;\beta_{k}x_{k}\;\ldots +\beta_{n}x_{n}$$

where *β*_k_ is the per-allele log OR for breast cancer associated with the minor allele for SNP *k*, and *x*_*k*_ the number of minor alleles for the same SNP (0, 1, or 2), and *n* = 313 is the total number of SNPs. 30 SNPs were excluded due to ambiguity, and 45 other SNPs were unmatched. As with the original study, the raw PGS of the European population was normalized to a standard deviation of 1 and a mean of 0. We then applied normalization using the average and SD of European samples to the remaining GIAs as done in prior studies testing the generalizability of PGS_313_.^10^^,^^11^

### Genotyping

Details of genotyping, imputation, and quality control procedures of our cohort have been previously described.^18^ For this study, variants that match the following 3 criteria were retained for PGS calculation: (1) a mean R2 imputation quality greater than 0.3 across genotype array batches, (2) a *p* value greater than 1 × 10^−6^ in ancestry-specific Hardy Weinberg equilibrium tests, and (3) a minor allele frequency greater than 0.005. We then performed linkage disequilibrium (LD) pruning with plink2 (--indep-pairwise 1000 50 0.05) and excluded the long-range LD regions. The top nine PCs were computed with the flashpca2 software.^14^

### Statistical methods

Logistic regression models were used to estimate the ORs for the PGS on breast cancer with age and the first nine principal components as covariates using the equation below:

(Equation 2) $$\log\left(\text{breastcancer}\right)=\beta_{0}+\beta_{1}\;\left(\text{PGS}\right)+\beta_{2}\;\left(\text{Age}\right)+\beta_{3}\;\left(\text{PC}1\right)+\ldots +\beta_{11}\;\left(\text{PC}9\right)$$

Rather than using age of diagnoses as per Mavaddat et al., we used the age at which an ICD-9 or ICD-10 corresponding to breast cancer appeared in the patient’s medical record. This is due to the fact that the age of diagnosis was not stored as structured data from our EHRs. We were also unable to include family history as a covariate for the same reason. To be consistent with Mavaddat et al., we also included the first nine principal components as covariates to account for potential differences in population structure across ancestries.

Logistic regression with the same covariates was used to estimate the ORs for breast cancer by deciles of the PGS, with the middle (50th percentile) as the reference. Percentiles and their ORs were calculated per GIA separately. To examine the discrimination of each PGS per GIA, we estimated the AUC using the standardized PGS score, age at biobanking, and first nine principal components as predictors.

### Survival analysis

Kaplan-Meier analysis was performed by using the PGS as a sole predictor for OS in days as a continuous variable. OS was calculated by subtracting death or present time from the first date at which an ICD-9 or ICD-10 code corresponding to breast cancer appeared in a patient’s medical record. Patients without OS values were discarded from the analysis (*N* = 12).

## Data and code availability

There are restrictions to the availability of dataset/code due to privacy concerns. De-identified individual-level data for UCLA ATLAS are available only to UCLA researchers and can be accessed through the Discovery Data Repository Dashboard (https://it.uclahealth.org/about/ohia/ohia-products/discovery-data-repository-dashboard-0). Summary ATLAS association statistics are publicly available at https://atlas-phewas.mednet.ucla.edu/. The PGS investigated in this manuscript is available at The PGS Catalog (PGP000001, https://www.pgscatalog.org/publication/PGP000001/).

## Supplemental information

Document S1. Figures S1‒S4, Tables S1‒S5, and supplemental materials and methods

Document S2. Article plus supplemental information
