# Supplementary material for: Generalizability of PGS313 for breast cancer risk in a Los Angeles biobank
Source: HGG Adv. 2024 May 3;5(3):100302. doi: 10.1016/j.xhgg.2024.100302 (PMC11137525; doi:10.1016/j.xhgg.2024.100302)
Supplement: Document S1. Figures S1‒S4, Tables S1‒S5, and supplemental materials and methods [file mmc1.pdf]

HGGA, Volume *5*

Supplemental information

Generalizability of PGS_{313} for breast cancer risk in a Los Angeles biobank

Helen Shang, Yi Ding, Vidhya Venkateswaran, Kristin Boulier, Nikhita Kathuria-Prakash, Parisa Boodaghi Malidarreh, Jacob M. Luber, and Bogdan Pasaniuc

Supplemental List

Figure S1: Kaplan Meier Curve for Overall Survival in European Patients with or without Chemotherapy Treatment

Figure S2: Kaplan Meier Curve for Overall Survival in European Patients above or below the 70th Percentile of PGS_{313}

Figure S3: Multivariable Cox Proportional Hazards Model on OS in European Patients

Figure S4: Genetic Admixture across GIAs

Table S1: Medications used for identifying HR+ breast cancer cases

Table S2: Medications used for identifying HER2+ breast cancer cases

Table S3: Association between PGS and Breast Cancer Risk in GIAs

Table S4: AUCs by GIA

Table S5: Downsampling Experiment

Supplemental Material and Methods

Supplemental References

Figure S1: Kaplan Meier Curve for Overall Survival in European Patients with or without Chemotherapy Treatment

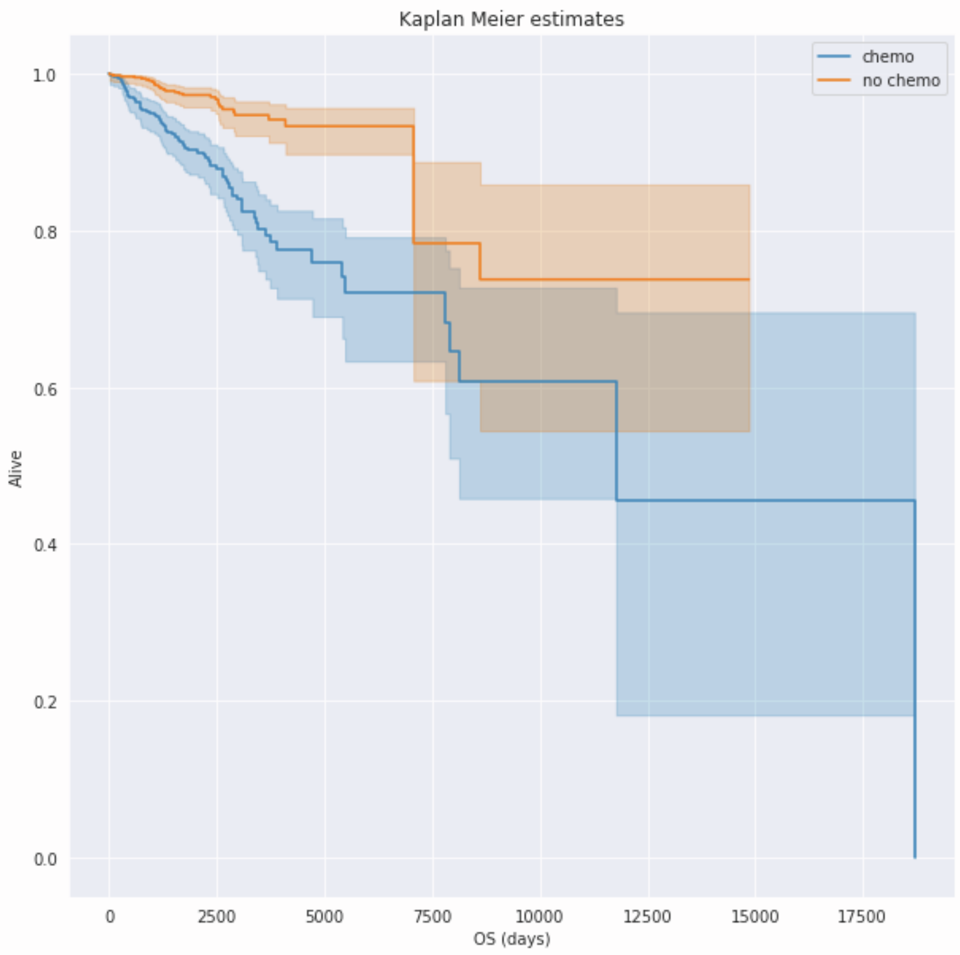

There were 503 patients who had received chemotherapy and 767 without chemotherapy in our European cohort. By Kaplan Meier survival analysis, we confirmed our approximate OS values were appropriately shorter in all patients who had received chemotherapies for breast cancer relative to those who had not (p value < .005). This is expected as patients who receive chemotherapy often have more aggressive or metastatic disease, resulting in shorter survival.

Figure S2: Kaplan Meier Curve for Overall Survival in European Patients above or below the 70th Percentile of PGS_{313}

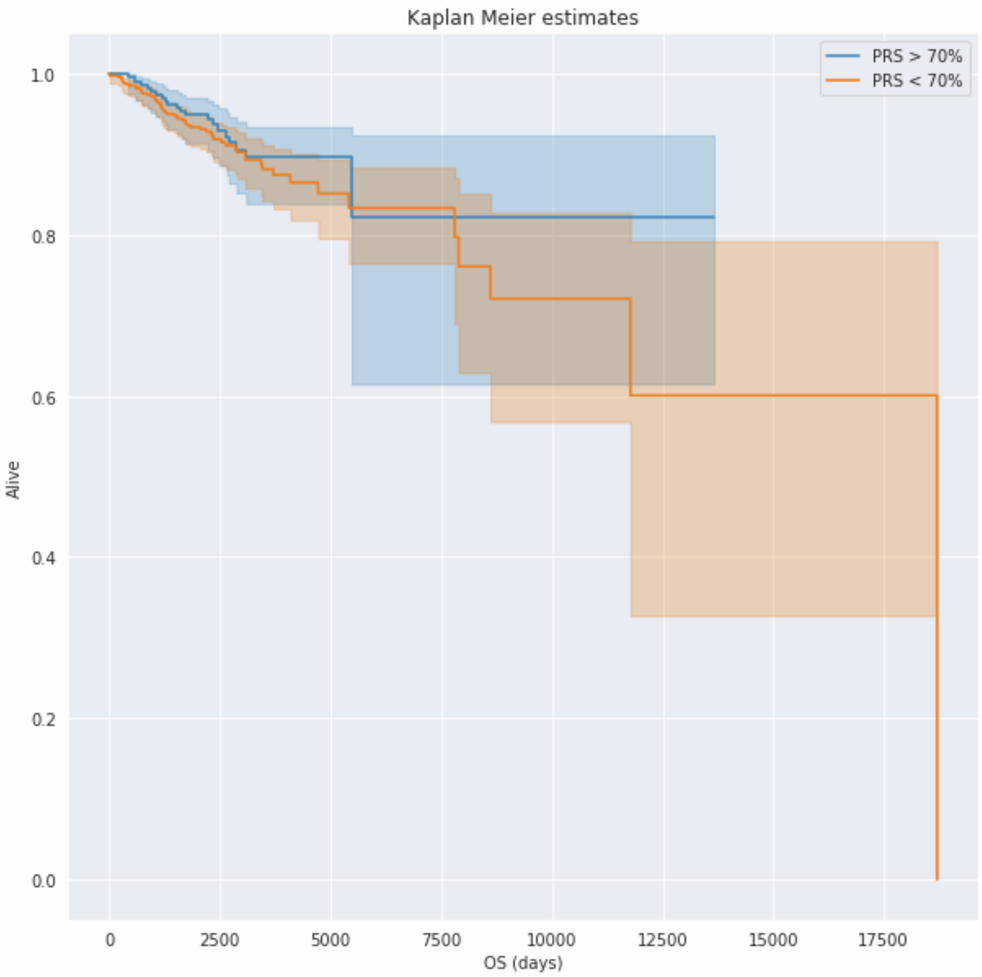

To evaluate the impact of PGS_{313} on OS, we compared survival times by Kaplan Meier analysis for European patients above (N=280) and below (N=651) the 70th percentile of the PGS. We found no difference in survival time between the two groups by log-rank test (p-value= 0.38). There was also no difference in survival time when comparing above and below the 50th percentile as well as the top 90th and lowest 10th percentiles.

Figure S3: Multivariable Cox Proportional Hazards Model on OS in European Patients

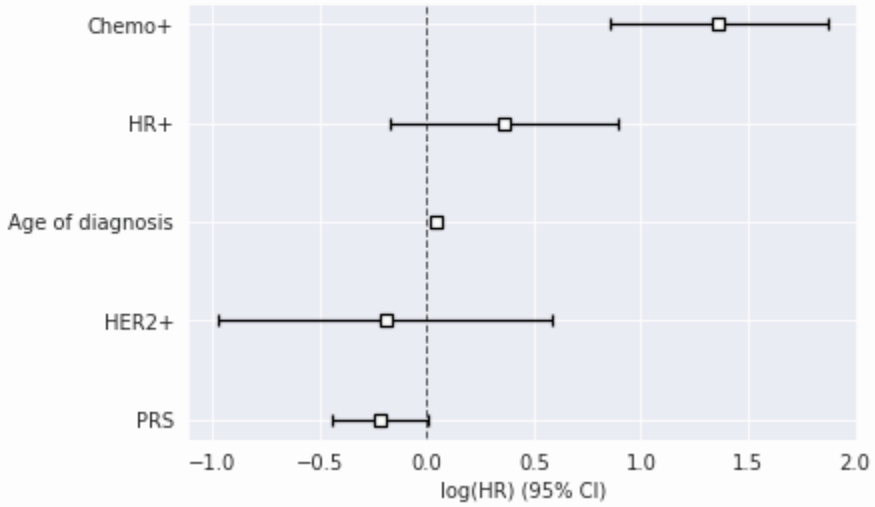

For European patients, we initially found by Cox Proportional Hazards that the normalized PGS_{313} was inversely predictive of OS, suggesting that a lower PGS_{313} score translates to longer survival time (HR, 0.80; 95 CI, 0.64-0.99, p-value = 0.04). However, when adjusted for other variables such as whether or not a patient had received chemotherapy, cancer subtype (HR+ and/or HER2+), and age of diagnosis, the normalized PGS_{313} score is no longer predictive of OS (p–value = 0.06).

Figure S4: Genetic Admixture across GIAs

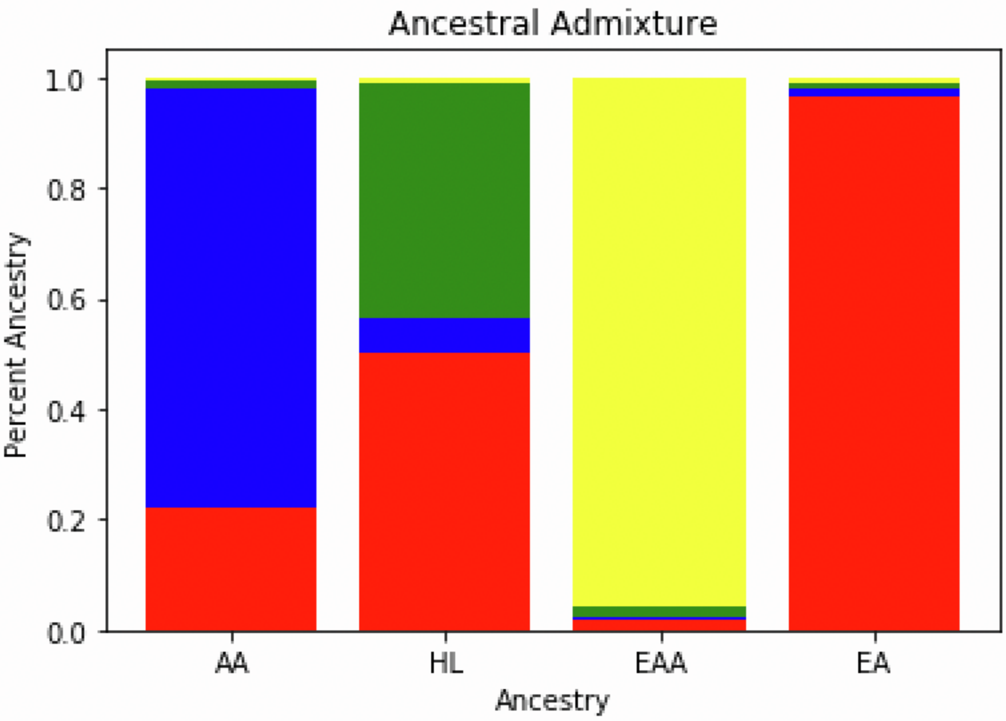

We found that genetic admixture was present but in varying degrees. The EAA population had the least overlap with the EA population, whereas the HL population had the most overlap with the EA population. These results suggest that genetic admixture may explain the overlapping performance of the PGS on our HL and EA cohorts.

Table S1: Medications used for identifying HR+ breast cancer

Name	Medication Class
Exemestane	Hormone therapy
Anastrazole	Hormone therapy
Letrozole	Hormone therapy
Tamoxifen	Hormone therapy
Raloxifine	Hormone therapy
Fulvestrant	Hormone therapy
Palbociclib	CDK 4/6 inhibitor
Ribociclib	CDK 4/6 inhibitor
Abemaciclib	CDK 4/6 inhibitor
Everolimus	mTOR inhibitor
Alpelisib	PI3K inhibitor

Table S2: Medications used for identifying HER2+ breast cancer

Name	Medication Class
Trastuzumab	HER2 monoclonal antibody
Pertuzumab	HER2 monoclonal antibody
Neratinib	Tyrosine Kinase Inhibitors
Lapatinib	Tyrosine Kinase Inhibitors
Ruxolitinib	Tyrosine Kinase Inhibitors
Osimertinib	Tyrosine Kinase Inhibitors
Tucatinib	Tyrosine Kinase Inhibitors
Niraparib	Tyrosine Kinase Inhibitors
Dasatinib	Tyrosine Kinase Inhibitors

Table S3: Association between PGS and Breast Cancer Risk in Genetically Inferred Ancestries (GIA)

GIA	OR	Lower CI	Upper CI
AA	1.31	1.05	1.64
EA	1.52	1.23	1.72
EAA	1.46	1.43	1.61
HL	1.51	1.31	1.75

Table S4: AUCs by Genetically-Inferred Ancestry

GIA	AUC	Lower CI	Upper CI
AA	0.613	0.567	0.659
EA	0.650	0.621	0.680
EAA	0.620	0.581	0.658
HL	0.688	0.676	0.700

We found overlapping AUCs for the HL and EA cohorts, as determined by their overlapping 95% confidence intervals. The AUCs for the AA and EAA populations were lower as determined by their non-overlapping 95% confidence intervals, relative to the EA and HL population.

Table S5: Downsampling Experiment

GIA	Batched OR	Batched Lower CI	Batched Higher CI	Raw OR
AA	1.40	0.73	2.07	1.31
EA	1.64	0.74	2.38	1.51
EAA	1.29	0.65	1.93	1.46
HL	1.69	0.97	2.66	1.52

To evaluate if differences in ORs were due to sample size imbalance across the GIAs, we conducted an ensemble downsampling of all groups in 500 batches. The averaged OR and 95% CI (see Batched OR and Batched CI) overlapped with all of the Raw ORs for each GIA, as determined by the overlapping 95% confidence intervals, suggesting that differences in OR observed in our AA cohort, relative to our EA cohort, are less likely due to differences in sample size. However, for the EA cohort, the batched 95% CI was wider than that observed for the raw data, suggesting that a component of the larger 95% CI spread for the AA cohort may be due to sample size, as expected.

Supplemental Material and Methods

Kaplan Meier Curves

We approximated Overall Survival (OS) in days by subtracting the present day or date of death from date of diagnosis amongst patients who were still living or dead, respectively, in the EHR. To confirm that our estimates of OS were reliable, we then compared survival time amongst these two groups by Kaplan Meier analysis to demonstrate that patients who had undergone chemotherapy had shorter survival times, as expected. Only European patients were chosen for this analysis due to fewer patients amongst other GIAs that had received chemotherapy. Kaplan Meier analysis was also used to evaluate survival time amongst European patients at the top 70th percentile of the PGS_{313} versus those below this threshold. The 70th percentile was chosen as starting at this threshold, the OR was noted to be statistically greater than 1 for the EA cohort (Figure 2).

Cox Proportional Hazards Model

Cox Proportional Hazards was used to model PGS_{313}'s effect in combination with other co-variates on OS. This would also allow us to confirm if there might be any cofounders of the $PGS_{313.}$ Due to limited cohort numbers, we chose to only model the European population, which had sufficient numbers of patients who had undergone chemotherapy and were HER2+. For our Cox Proportional Hazards model, we included whether or not a patient had received chemo, HER2 status, normalized PGS_{313} and our estimated age

at diagnosis based on when an ICD code related to breast cancer first appeared in the EHR.

Subtyping

We first queried the top 50 most commonly prescribed cancer-related medications within the electronic medical record for our cohort in the EHR. These were manually reviewed and grouped based on class and subtype relevance. Supplemental Table 1 shows our grouped list of HR+ relevant medications and Supplemental Table 2 shows our grouped list of HER2+ relevant medications. Patients were subtyped as HR+ and/or HER2+ if they had been ordered at least one relevant medication from each list in the EHR.

Genetic Admixture

Please refer to our prior publication regarding our methodology for our genetic admixture analysis using k=4 subpopulations, representing the number of genetically inferred ancestries (GIAs) in our study[1].

Association between PGS and Breast Cancer Risk in Genetically Inferred Ancestries (GIA)

Univariate logistic regression was used to evaluate the association between PGS_{313} and observed rates of all breast cancers in American females of African (AA), European (EA), East Asian American (EAA), and Hispanic (HL) genetically inferred ancestry. As with prior studies testing the generalizability of PGS_{313}, we normalized the raw PGS

score of non-European GIAs based on the average and standard deviation of European samples. In comparison, Mavaddat et al. reported an OR of 1.61 (95 CI 1.57-1.65), which overlaps with all GIAs.

AUCs by Genetically-Inferred Ancestry

Logistic regression was performed to predict the labeling of breast cancer status using the normalized PGS, Age at diagnosis, and PCs 1-9 as co-variates, as consistent with Mavaddat et al. AUCs were calculated for each of the four different GIAs separately.

Downsampling experiment

To confirm that differences in ORs were not due to sample size imbalance across the GIAs, we conducted an ensemble downsampling of all groups such that all were downsampled into 500 batches of 124 cases and 124 controls each, which were randomly selected, as this was the number of cases in the AA cohort, which had the fewest number of cases across all GIAs. We then calculated the average OR and 95% CI across all 500 experiments (see Batched OR and Batched CI) and compared this with the Raw ORs for each GIA.

Supplemental References

1. Johnson R, Ding Y, Venkateswaran V, et al. (2022). Leveraging genomic diversity for discovery in an electronic health record linked biobank: the UCLA ATLAS Community Health Initiative. Genome Med *1*, 104. 10.1186/s13073-022-01106-x.
