# Supplementary material for: Generalizability of PGS313 for breast cancer risk in a Los Angeles biobank
Source: HGG Adv. 2024 May 3;5(3):100302. doi: 10.1016/j.xhgg.2024.100302 (PMC11137525; doi:10.1016/j.xhgg.2024.100302)
Supplement: Document S2. Article plus supplemental information [file mmc2.pdf]

HGG Advances

ARTICLE

Generalizability of PGS_{313} for breast cancer risk in a Los Angeles biobank

Helen Shang,[1,2,15,*] Yi Ding,[3] Vidhya Venkateswaran,[4] Kristin Boulier,[5] Nikhita Kathuria-Prakash,[6] Parisa Boodaghi Malidarreh,[7,8] Jacob M. Luber,[7,8,9] and Bogdan Pasaniuc[10,11,12,13,14]

Summary

Polygenic scores (PGSs) summarize the combined effect of common risk variants and are associated with breast cancer risk in patients without identifiable monogenic risk factors. One of the most well-validated PGSs in breast cancer to date is PGS_{313}, which was developed from a Northern European biobank but has shown attenuated performance in non-European ancestries. We further investigate the generalizability of the PGS_{313} for American women of European (EA), African (AFR), Asian (EAA), and Latinx (HL) ancestry within one institution with a singular electronic health record (EHR) system, genotyping platform, and quality control process. We found that the PGS_{313} achieved overlapping areas under the receiver operator characteristic (ROC) curve (AUCs) in females of HL (AUC = 0.68, 95% confidence interval [CI] = 0.65–0.71) and EA ancestry (AUC = 0.70, 95% CI = 0.69–0.71) but lower AUCs for the AFR and EAA populations (AFR: AUC = 0.61, 95% CI = 0.56–0.65; EAA: AUC = 0.64, 95% CI = 0.60–0.680). While PGS_{313} is associated with hormone-receptor-positive (HR+) disease in EA Americans (odds ratio [OR] = 1.42, 95% CI = 1.16–1.64), this association is lost in African, Latinx, and Asian Americans. In summary, we found that PGS_{313} was significantly associated with breast cancer but with attenuated accuracy in women of AFR and EAA descent within a singular health system in Los Angeles. Our work further highlights the need for additional validation in diverse cohorts prior to the clinical implementation of PGSs.

Introduction

The US Preventive Services Task Force recommends that breast cancer screening start at 50 years old, based on studies showing that 90% of breast cancer cases are diagnosed after this age.[1] Unfortunately, this also means that 10% of cases will be missed per conventional guidelines, equating to approximately 10,000 missed cases in the US annually.[2] As such, researchers have been working to develop new methods of identifying patients at risk of developing early-onset breast cancer. One well-known approach is the Gail model, which uses clinical, family history, and demographic information to calculate individual breast cancer risk but suffers from poor accuracy; in a meta-analysis across 26 studies and 29 datasets, a modified version of the Gail model had areas under the receiver operator characteristic (ROC) curve (AUCs) for American, Asian, and European females of 0.61 (95% confidence interval [CI] = 0.59–0.63), 0.55 (95% CI = 0.52–0.58), and 0.58 (95% CI = 0.55–0.62), respectively.[3,4]

Another avenue has been identifying carriers of genes such as *BRCA1*, *BRCA2*, *PTEN*, *TP53*, *CDH1*, and *STK11*, which are associated with a 2- to 3-fold elevated risk in developing breast cancer over a patient's lifetime.[5] However, the vast majority (>75%) of patients with breast cancer do not have any identifiable monogenic risk factors and thus will not benefit from this approach. Over the past decade, polygenic scores (PGSs) were introduced as a potential solution by summarizing the combined effect of multiple common risk variants that have been individually associated with small yet elevated breast cancer risk.[6] Several PGSs have been developed to predict and stratify breast cancer risk, including a recent paper showing that at the top 5th percentile of one PGS had a genetic risk of similar magnitude to some monogenic etiologies.[7] Prior work has estimated that the theoretically best PGS, if the effect sizes of all common SNPs were known with certainty, would explain ~41% of the familial risk of breast cancer.[8]

One of the most validated PGSs in breast cancer to date is the PGS_{313} by Mavaddat et al., which was developed from a

[1]Division of Internal Medicine, Ronald Reagan UCLA Medical Center, Los Angeles, CA, USA; [2]Department of Computer Science and Engineering, University of Texas at Arlington, Arlington, TX, USA; [3]Bioinformatics Interdepartmental Program, University of California, Los Angeles, Los Angeles, CA, USA; [4]Department of Oral Biology, UCLA School of Dentistry, Los Angeles, CA, USA; [5]Division of Cardiology, Department of Medicine, Ronald Reagan UCLA Medical Center, Los Angeles, CA, USA; [6]Division of Hematology-Oncology, Department of Medicine, Ronald Reagan UCLA Medical Center, Los Angeles, CA, USA; [7]Department of Computer Science and Engineering, University of Texas at Arlington, Arlington, TX, USA; [8]Multi-Interprofessional Center for Health Informatics, University of Texas at Arlington, Arlington, TX, USA; [9]Department of Bioengineering, University of Texas at Arlington, Arlington, TX, USA; [10]Bioinformatics Interdepartmental Program, University of California, Los Angeles, Los Angeles, CA, USA; [11]Department of Pathology and Laboratory Medicine, David Geffen School of Medicine, University of California, Los Angeles, Los Angeles, CA, USA; [12]Department of Human Genetics, David Geffen School of Medicine, University of California, Los Angeles, Los Angeles, CA, USA; [13]Department of Computational Medicine, David Geffen School of Medicine, University of California, Los Angeles, Los Angeles, CA, USA; [14]Institute of Precision Health, University of California, Los Angeles, Los Angeles, CA, USA
[15]Lead contact
*Correspondence: hshang@mednet.ucla.edu
https://doi.org/10.1016/j.xhgg.2024.100302.
This is an open access article under the CC BY license (http://creativecommons.org/licenses/by/4.0/).

Table 1. Participant characteristics

	AA	EA	EAA	HL
Controls	1,032	10,407	1,803	3,083
Cases (% of total)	124 (10)	1,466 (12)	207 (10)	220 (6.7)
Age at diagnosis, mean (SD)	55 (17)	55 (17)	52 (17)	48 (17)
Cases with prescription data (% of total)	86 (52)	1,241 (58)	188 (64)	166 (58)
HR+ (% of cases with prescription data)	60 (71)	979 (72)	129 (65)	115 (65)
HER2+ (% of cases with prescription data)	11 (12)	130 (10)	26 (14)	18 (11)
OS in days, mean (SD)	2,862 (1,922)	2,741 (1,973)	2,569 (1,427)	2,911 (2,149)

Participants were females drawn from the UCLA ATLAS Biobank ($N = 18{,}627$), which is linked to UCLA medical records from 2013 to present day. Cases and controls were identified based on ICD-9 and ICD-10 coding corresponding to breast cancer. Age at diagnosis was based on the date at which the ICD code appeared in a patient's chart, which was then used to calculate the overall survival (OS), with the day of death or present day as an endpoint. Breast cancer subtypes were identified based on prescriptions ordered.

Northern European biobank (N = 33,673 cases and N = 33,381 controls). When incorporating family history and age of diagnosis, the PGS_{313} achieved an odds ratio (OR) of 1.61 (95% CI = 1.57–1.65) and an AUC of 0.63 (95% CI = 0.63–0.65).[9] Subsequent work has demonstrated an attenuated effect of PGS_{313} in African females; for example, Cong et al. found that for 33,594 women of European ancestry (EA) and 2,801 women of African (AFR) ancestry (AA) across 9 institutions, the PGS_{313} alone achieved a higher AUC for European females (0.60, 95% CI = 0.59–0.61) than for AFR females (0.55, 95% CI = 0.51–0.58).[10,11]

In this paper, we aim to further investigate the generalizability of the PGS_{313} for American women of AFR, Asian, and Latinx ancestry within one institution, leveraging a singular electronic health record (EHR) system, genotyping platform, and quality control process.

Results

Our study included 18,627 women, including 1,156 with AA, 11,873 women with EA, 2,010 with East Asian ancestry (EAA), and 3,303 with Hispanic ancestry (HL), as presented in Table 1. The majority of cases were identified as hormone-receptor positive (HR+) (AA: 71%; EA: 72%; EAA: 65%; HL: 65%), and the prevalence across ancestries was comparable to the latest SEER registry showing that 70% of breast cancer subtypes are HR+.[12] Similarly, we found prevalences of human epidermal growth factor receptor 2-positive (HER2+) disease that were also comparable to SEER registries showing a prevalence of 10.8%. For 1,285 out of 2,080 cases, we did not have any prescription data, as these patients may have undergone cancer treatment outside of the UCLA network.

Association of PGS_{313} with breast cancer risk in various ancestries

For each genetically inferred ancestry (GIA) group, the PGS followed a normalized distribution with the EAA cohort having a higher mean, as illustrated in Figure 1 (AA: N = 1,156, mean = –0.02, SD = 0.46; EA: N = 11,873, mean = –0.06, SD = 0.53; EAA: N = 2,010, mean = 0.15, SD = 0.47; HL: N = 3,303, mean = –0.06, SD = 0.53). We found statistically significant associations of PGS_{313} with overall breast cancer risk across all ancestries. All GIAs had overlapping ORs (AA: OR = 1.31, 95% CI = 1.05–1.64; EA: OR = 1.52, 95% CI = 1.23–1.72; EAA: OR = 1.46, 95% CI = 1.23–1.61; HL: OR = 1.51, 95% CI = 1.31–1.75). These also overlap with Mavaddat et al.'s reported OR in Europeans (OR = 1.61, 95% CI = 1.57 to 1.65).

As seen in Figure 2, for all GIAs, the ORs of the PGS_{313} was largest at the extremes PGS distribution and the 95% CIs overlap among the four GIAs (AA: OR = 1.85, 95% CI = 1.08–3.2; EA: OR = 2.1, 95% CI = 1.9–2.6; EAA: OR = 1.7, 95% CI = 1.1–2.6; HL: OR = 1.7, 95% CI = 1.1–2.5). For the AA population, we found an attenuated effect of the PGS and at the extremes of its distribution (>95th percentile) relative to other GIAs, although this analysis was limited by small sample sizes (data not shown).

To confirm that differences in ORs were not due to sample size imbalances, we conducted an ensemble downsampling experiment. All GIAs were downsampled into 500 batches consisting of 124 cases and 124 controls each, which were randomly selected, as this was the number of cases in the AA cohort, which had the fewest. As shown in Table S4, for each GIA, the batched OR overlapped with the raw OR, suggesting that differences in raw ORs across GIAs are less likely due to differences in sample size.

Discriminative accuracy of the PGS_{313}

The discriminative accuracy of the PGS for any type of breast cancer, as measured by the AUC, was highest in the EA population at 0.70 (95% CI = 0.69–0.71), which is slightly higher than 0.63 (95% CI = 0.63–0.65) as reported by Mavaddat et al. Relative to the EA population, the AUC for the HL population was similar, whereas those for the AFR and EAA populations were lower (AFR: AUC = 0.61, 95% CI = 0.56–0.65; EAA: AUC = 0.64, 95% CI = 0.60–0.680; HL: AUC = 0.68, 95% CI = 0.65–0.71). There was no statistical difference in the AUCs for the EA and HL populations (HL: AUC = 0.68, 95% CI = 0.65–0.71),

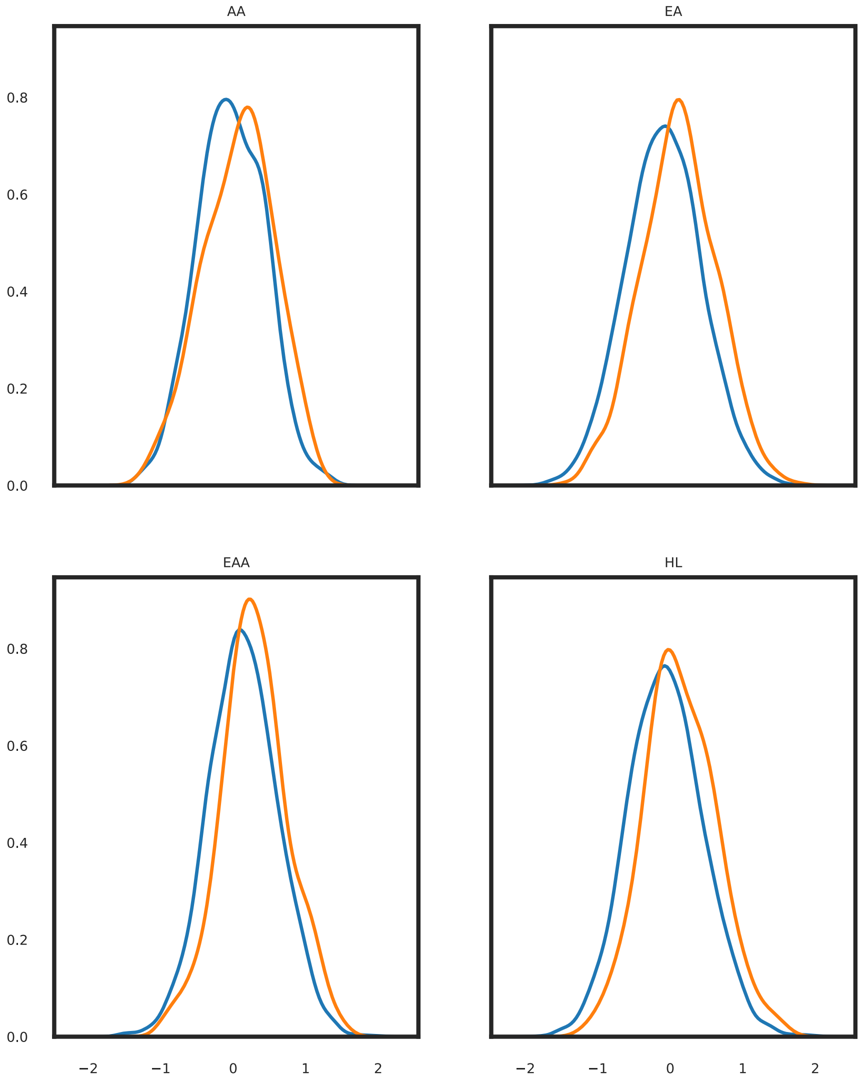

Figure 1. Distribution of PGS_{313} in cases and controls
Kernel distribution estimation plots of PGS_{313} scores in cases and controls by genetically inferred ancestry (GIA). The orange curves represent scores for cases and blue curves represent scores for controls. The raw PGS_{313} scores of the European population (European ancestry [EA]) was normalized to a standard deviation of 1 and a mean of 0. The remaining GIAs were normalized to the average and standard deviation of EA samples.

as determined by their overlapping 95% CIs. In contrast, the AFR and EAA populations were statistically different, as determined by their lower AUCs and non-overlapping 95% CIs, relative to the EA and HL populations (AFR: AUC = 0.61, 95% CI = 0.56–0.65; EAA: AUC = 0.64, 95% CI = 0.60–0.680).

Association of PGS_{313} with breast cancer subtypes in various ancestries

Recent work in European females has shown that HR+ breast cancer is associated with PGS_{313} and a lower risk genetic signature.[13] As with prior work, we found that for European patients, the PGS_{313} was associated with HR+ disease; however, it was not associated with HR+ disease in Hispanic, AFR, or Asian American females with breast cancer (Table 2). In contrast, among Hispanic Americans alone, HER2+ disease was instead associated with PGS_{313} with a higher OR than for all breast cancer risk (OR = 2.47, 95% CI = 1.39–4.41), although this analysis was limited, as there were only 18 patients within our cohort that were both Hispanic and HER2+.

Survival analysis

To confirm that our estimates of overall survival (OS) were reliable, we first confirmed that OS is appropriately associated with whether or not a patient had received chemotherapy, as a hallmark of more aggressive and often metastatic disease (Figure S1). We found that in European patients, the PGS_{313} alone as a predictor fails to stratify patients by survival time by Kaplan-Meier analysis when comparing patients with breast cancer above ($N = 280$) or below ($N = 651$) the 70th percentile with a log-rank p value of 0.38 (Figure S2A). We chose to compare above and below the 70th percentile of PGS313 as, starting at this threshold, the OR was noted to be statistically greater than 1 for the EA cohort, as seen in Figure 2. Of note, we found similar results when evaluating different thresholds, such as comparing the top vs. bottom 50th percentiles and the top 90th vs. bottom 10th percentiles. For European patients, after accounting for whether a patient had received chemotherapy, age of diagnosis, HER2+, and PR+ disease using a multivariate Cox proportional hazard model, PGS_{313} was also no longer predictive of OS (Figure S2B). This is consistent with recent work involving a cohort of European (N = 98,397) and Asian (N = 12,920) females with breast cancer, which found that in European patients, PGS_{313} was no longer associated with OS after adjusting for breast cancer subtype and tumor grade.[13]

Discussion

In this paper, we investigated the generalizability of the PGS_{313} for American women of AFR, Asian, and Latinx ancestry within one institution. For females of EA, we arrived at overlapping estimates of OR (1.5; 95% CI = 1.2–1.7) when compared to Mavaddat et al. (1.61; 95% CI = 1.57–1.65). Consistent with prior studies, we found that the PGS_{313} was still associated with breast cancer

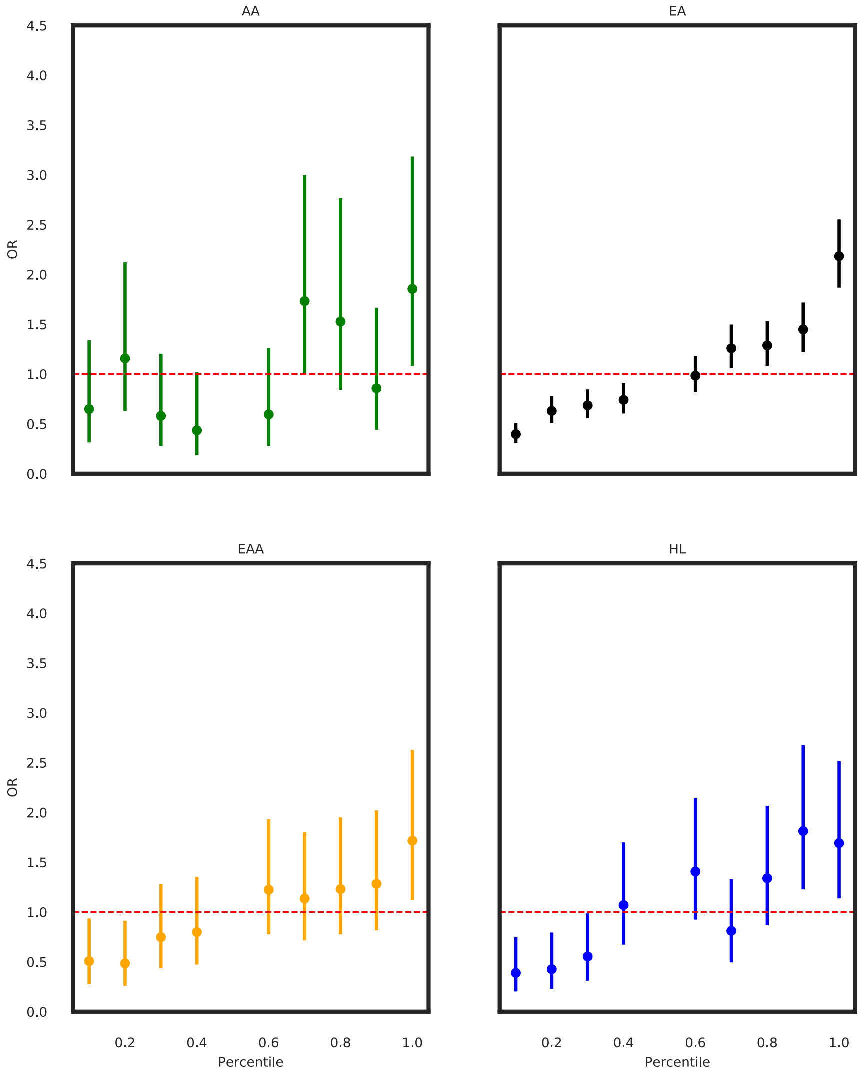

Figure 2. Association of PGS_{313} deciles with breast cancer relative to the 50th percentile
Association between PGS_{313} and breast cancer diagnoses in American women of African (AA), European (EA), East Asian American (EAA), and Hispanic (HL) ancestry, based on GIAs, where the OR is plotted on the y axis and percentiles of the PGS_{313} are plotted on the x axis. ORs and 95% confidence intervals are shown. ORs are for different deciles of the PGS relative to the 50th percentile of the PGS.

across all ancestries but with an attenuated effect in females of AFR and Asian ancestry; the PGS_{313} achieved equivalent AUCs in females of Latinx ancestry (AUC = 0.68, 95% CI = 0.65–0.71) and EA (AUC = 0.70, 95% CI = 0.69–0.71), with lower AUCs for the AFR and EAA populations (AFR: AUC = 0.61, 95% CI = 0.56–0.65; EAA: AUC = 0.64, 95% CI = 0.60–0.680). This may be due to Latinx individuals having a greater proportion of EA ancestry than Asians and AFR individuals, as we found that the HL cohort had higher levels of EA ancestry relative to all other GIAs (Figure S4).

Consistent with prior work, we found that for European American patients, PGS_{313} is associated with HR+ disease.[9,13] For AFR, Hispanic, and Asian American patients, this association is lost, although rates of HR+ disease were lower in these cohorts relative to European Americans. While we unexpectedly found that for Hispanic Americans alone, the PGS_{313} was associated with HER2+ disease, given our cohort's limited size, further investigation regarding this association is warranted.

Similarly, as with recent work on PGS_{313}, we found that it fails to stratify European patients by OS.[14] One possible explanation is that the impact of PGS_{313} on OS may be confounded by other genetic risk factors, many of which have yet to be identified; several recent papers have found that PGS_{313} stratifies breast cancer risk in *CHEK2*, *PALB2*, and *ATM* carriers but not *BRCA1*/2 carriers.[15,16] In other words, the value of PGS_{313} may be in stratifying carriers of low-penetrance risk variants but may fail to stratify those with highly penetrant variants who will go on to develop breast cancer regardless. While we were not able to confirm this in our study given the few number of risk carriers in our cohort, we hope to validate this in future studies.

There are many limitations of this study to consider. Our cohort contained fewer non-European than European samples, and thus analyses at the upper extremes and within subtypes were limited. We also could not estimate the absolute risk of developing breast cancer due to the lack of longitudinal outcomes data. Furthermore, many covariates such as age of diagnoses, cancer subtype, and OS were not available in the medical record as structured data and were thus calculated by proxy methods. Nevertheless, we were able to confirm that these estimates resulted in expected observations, suggesting their reliability, such as the expected prevalence rates among breast cancer subtypes and shorter survival times for patients recieving chemotherapy versus those without due to more aggressive disease.

In summary, we found that PGS_{313} was significantly associated with breast cancer in American females of diverse ancestries but with attenuated accuracy in women of AFR and Asian descent within a singular yet diverse biobank in Los Angeles. While the PGS_{313} is associated with HR+ disease in European Americans, this association is lost in AFR, Hispanic, and Asian Americans. For Hispanic Americans, PGS_{313} may be instead associated with HER2+ disease, although due to small numbers, additional studies will be critical in validating these findings. Our

Table 2. Association of PRS_{313} with breast cancer with HR+ and HER2+ disease by genetically inferred ancestry

	HR−	HR+	OR	Lower CI	Upper CI
AA	15	51	0.71	0.36	1.40
EA	176	768	1.42	1.16	1.64
EAA	22	114	1.38	0.41	1.20
HL	33	106	0.70	0.91	2.20
	HER2−	HER2+	OR	Lower CI	Upper CI
AA	55	11	2.26	0.96	5.33
EAS	814	130	0.95	0.79	1.15
EAA	110	26	1.02	0.62	1.66
HL	121	18	2.47	1.39	4.41

The top section shows observed ORs for the PGS_{313} in predicting hormone-receptor-positive (HR+) or hormone-receptor-negative (HR−) breast cancer across the four GIAs. Logistic regression was performed to predict the labeling of specific subtypes among patients with breast cancer only. The bottom section shows observed ORs for the PGS_{313} in predicting HER+ or HER2− breast cancer across the four GIAs. Columns 2 and 3 reflect positive cases (HR+/HER2+) and negative cases (HR−/HER2−), respectively. PGS313 is only predictive of HR+ disease in European American patients ($p = 0.0002$) but not other ancestries. PGS_{313} is only statistically significant in predicting HER2+/− disease for the Hispanic American cohort, as indicated by the OR's 95% CI not crossing 1.

work further highlights the need for additional validation in diverse cohorts prior to clinical implementation of PGSs and the need for new methods that can address differences in genomic admixture.

Subjects and methods

Study participants

The participants included in this cohort study were females at birth drawn from the UCLA ATLAS Biobank ($N = 18{,}627$), which is linked to electronic medical record data and has been described previously.[17] SNPs were genotyped on a genome-wide array and imputed to the TOPmed reference panel. We identified breast cancer cases and controls using ICD-9 and ICD-10 codes corresponding to Phecode X 105.1, specifying "malignant neoplasm of the breast, female," which maps ICD codes to clinically meaningful phenotypes.[17] Patient recruitment and sample collection has been approved by the UCLA Insitutional Review Board (UCLA IRB) IRB#17-001013.

We previously identified five GIAs based on principal-component analysis and k-means clustering including African Americans, Hispanic Latino Americans, East Asian Americans, European Americans, and South Asian Americans (SAA).[18] The SAA population was not included for further analysis due to case counts being significantly underpowered. As we did not have access to individual-level pathology results, we identified HR+ or HER2+ breast cancer based on prescription data correlating to either subtype.

PGS models

The PGS was calculated based on an additive model using effect size estimates from the PGS as initially developed by Mavaddat et al., which are available under the entry PGS000004 within The PGS Catalog:[19]

$$PGS = \beta_1 x_1 + \beta_2 x_2 + \ldots \beta_k x_k \ldots + \beta_n x_n \qquad \text{(Equation 1)}$$

where β_k is the per-allele log OR for breast cancer associated with the minor allele for SNP k, and x_k the number of minor alleles for the same SNP (0, 1, or 2), and $n = 313$ is the total number of SNPs. 30 SNPs were excluded due to ambiguity, and 45 other SNPs were unmatched. As with the original study, the raw PGS of the European population was normalized to a standard deviation of 1 and a mean of 0. We then applied normalization using the average and SD of European samples to the remaining GIAs as done in prior studies testing the generalizability of PGS_{313}.[10,11]

Genotyping

Details of genotyping, imputation, and quality control procedures of our cohort have been previously described.[18] For this study, variants that match the following 3 criteria were retained for PGS calculation: (1) a mean R2 imputation quality greater than 0.3 across genotype array batches, (2) a p value greater than 1×10^{-6} in ancestry-specific Hardy Weinberg equilibrium tests, and (3) a minor allele frequency greater than 0.005. We then performed linkage disequilibrium (LD) pruning with plink2 (–indep-pairwise 1000 50 0.05) and excluded the long-range LD regions. The top nine PCs were computed with the flashpca2 software.[14]

Statistical methods

Logistic regression models were used to estimate the ORs for the PGS on breast cancer with age and the first nine principal components as covariates using the equation below:

$$\log(\text{breastcancer}) = \beta_0 + \beta_1\,(\text{PGS}) + \beta_2\,(\text{Age}) + \beta_3\,(\text{PC1}) + \ldots + \beta_{11}\,(\text{PC9}) \qquad \text{(Equation 2)}$$

Rather than using age of diagnoses as per Mavaddat et al., we used the age at which an ICD-9 or ICD-10 corresponding to breast cancer appeared in the patient's medical record. This is due to the fact that the age of diagnosis was not stored as structured data from our EHRs. We were also unable to include family history as a covariate for the same reason. To be consistent with Mavaddat et al., we also included the first nine principal components as covariates to account for potential differences in population structure across ancestries.

Logistic regression with the same covariates was used to estimate the ORs for breast cancer by deciles of the PGS, with the middle (50th percentile) as the reference. Percentiles and their ORs were calculated per GIA separately. To examine the discrimination of each PGS per GIA, we estimated the AUC using the standardized PGS score, age at biobanking, and first nine principal components as predictors.

Survival analysis

Kaplan-Meier analysis was performed by using the PGS as a sole predictor for OS in days as a continuous variable. OS was calculated by subtracting death or present time from the first date at which an ICD-9 or ICD-10 code corresponding to breast cancer appeared in a patient's medical record. Patients without OS values were discarded from the analysis ($N = 12$).

Data and code availability

There are restrictions to the availability of dataset/code due to privacy concerns. De-identified individual-level data for UCLA ATLAS are available only to UCLA researchers and can be accessed through the Discovery Data Repository Dashboard (https://it.uclahealth.org/about/ohia/ohia-products/discovery-data-repository-dashboard-0). Summary ATLAS association statistics are publicly available at https://atlas-phewas.mednet.ucla.edu/. The PGS investigated in this manuscript is available at The PGS Catalog (PGP000001, https://www.pgscatalog.org/publication/PGP000001/).

Supplemental information

Supplemental information can be found online at https://doi.org/10.1016/j.xhgg.2024.100302.

Acknowledgments

We gratefully acknowledge the Institute for Precision Health, participating patients from the UCLA ATLAS Precision Health Biobank, the UCLA David Geffen School of Medicine, the UCLA Clinical and Translational Science Institute, and UCLA Health. V.V. is funded by NIH/NIDCR 5K12DE027830-04. The ATLAS Community Health Initiative is supported by UCLA Health, the David Geffen School of Medicine, and a grant from the UCLA Clinical and Translational Science Institute (UL1TR001881). B.P. was partially supported by NIH awards R01 HG009120, R01 MH115676, R01 CA251555, R01 AI153827, R01 HG006399, R01 CA244670, and U01 HG011715. K.B. is supported by UCLA T32 funding. J.M.L. is supported by the Cancer Prevention Research Institute of Texas and the University of Texas Rising STAR Award.

Declaration of interests

The authors declare no competing interests.

Received: May 5, 2023
Accepted: April 30, 2024

References

1. Siu, A.L.; and U.S. Preventive Services Task Force (2016). Screening for Breast Cancer: U.S. Preventive Services Task Force Recommendation Statement. Ann. Intern. Med. *164*, 279–296. https://doi.org/10.7326/M15-2886.
2. Acheampong, T., Kehm, R.D., Terry, M.B., Argov, E.L., and Tehranifar, P. (2020). Incidence Trends of Breast Cancer Molecular Subtypes by Age and Race/Ethnicity in the US From 2010 to 2016. JAMA Netw. Open *8*, e2013226. https://doi.org/10.1001/jamanetworkopen.2020.
3. Gail, M.H., Brinton, L.A., Byar, D.P., Corle, D.K., Green, S.B., Schairer, C., and Mulvihill, J.J. (1989). Projecting individualized probabilities of developing breast cancer for white females who are being examined annually. J. Natl. Cancer Inst. *81*, 1879–1886. https://doi.org/10.1093/jnci/81.24.1879.
4. Wang, X., Huang, Y., Li, L., Dai, H., Song, F., and Chen, K. (2018). Assessment of performance of the Gail model for predicting breast cancer risk: a systematic review and meta-analysis with trial sequential analysis. Breast Cancer Res. *20*, 18. https://doi.org/10.1186/s13058-018-0947-5.
5. Shiovitz, S., and Korde, L.A. (2015). Genetics of breast cancer: a topic in evolution. Ann. Oncol. *26*, 1291–1299. https://doi.org/10.1093/annonc/mdv022.
6. Yanes, T., Young, M.A., Meiser, B., and James, P.A. (2020). Clinical applications of polygenic breast cancer risk: a critical review and perspectives of an emerging field. Breast Cancer Res. *22*, 21. https://doi.org/10.1186/s13058-020-01260-3.
7. Jia, G., Lu, Y., Wen, W., Long, J., Liu, Y., Tao, R., Li, B., Denny, J.C., Shu, X.O., and Zheng, W. (2020). Evaluating the Utility of Polygenic Risk Scores in Identifying High-Risk Individuals for Eight Common Cancers. JNCI Cancer Spectr. *4*, pkaa021. https://doi.org/10.1093/jncics/pkaa021.
8. Michailidou, K., Lindström, S., Dennis, J., Beesley, J., Hui, S., Kar, S., Lemaçon, A., Soucy, P., Glubb, D., Rostamianfar, A., et al. (2017). Association analysis identifies 65 new breast cancer risk loci. Nature *551*, 92–94. https://doi.org/10.1038/nature24284.
9. Mavaddat, N., Michailidou, K., Dennis, J., Lush, M., Fachal, L., Lee, A., Tyrer, J.P., Chen, T.H., Wang, Q., Bolla, M.K., et al. (2019). Polygenic risk scores for prediction of breast cancer and breast cancer subtypes. Am. J. Hum. Genet. *104*, 21–34. https://doi.org/10.1016/j.ajhg.2018.11.002.
10. Liu, C., Zeinomar, N., Chung, W.K., Kiryluk, K., Gharavi, A.G., Hripcsak, G., Crew, K.D., Shang, N., Khan, A., Fasel, D., et al. (2021). Generalizability of Polygenic Risk Scores for Breast Cancer Among Women With European, African, and Latinx Ancestry. JAMA Netw. Open *4*, e2119084. https://doi.org/10.1001/jamanetworkopen.2021.19084.
11. Du, Z., Gao, G., Adedokun, B., Ahearn, T., Lunetta, K.L., Zirpoli, G., Troester, M.A., Ruiz-Narváez, E.A., Haddad, S.A., PalChoudhury, P., et al. (2021). Evaluating Polygenic Risk Scores for Breast Cancer in Women of African Ancestry. J. Natl. Cancer Inst. *113*, 1168–1176. https://doi.org/10.1093/jnci/djab050.
12. Howlader, N., Altekruse, S.F., Li, C.I., Chen, V.W., Clarke, C.A., Ries, L.A.G., and Cronin, K.A. (2014). US incidence of breast cancer subtypes defined by joint hormone receptor and HER2 status. J. Natl. Cancer Inst. *106*, dju055. https://doi.org/10.1093/jnci/dju055.
13. Lopes Cardozo, J.M.N., Andrulis, I.L., Bojesen, S.E., Dörk, T., Eccles, D.M., Fasching, P.A., Hooning, M.J., Keeman, R., Nevanlinna, H., Rutgers, E.J.T., et al. (2023). Associations of a Breast Cancer Polygenic Risk Score With Tumor Characteristics and Survival. J. Clin. Oncol. *10*, 1849–1863. https://doi.org/10.1200/JCO.22.01978.
14. Abraham, G., and Inouye, M. (2014). Fast principal component analysis of large-scale genome-wide data. PLoS One *9*, e93766. https://doi.org/10.1371/journal.pone.0093766.
15. Gallagher, S., Hughes, E., Wagner, S., Tshiaba, P., Rosenthal, E., Roa, B.B., Kurian, A.W., Domchek, S.M., Garber, J., Lancaster, J., et al. (2020). Association of a Polygenic Risk Score

With Breast Cancer Among Women Carriers of High- and Moderate-Risk Breast Cancer Genes. JAMA Netw. Open *3*, e208501. https://doi.org/10.1001/jamanetworkopen.2020.8501.

16. Lakeman, I.M.M., Rodríguez-Girondo, M.D.M., Lee, A., Celosse, N., Braspenning, M.E., van Engelen, K., van de Beek, I., van der Hout, A.H., Gómez García, E.B., Mensenkamp, A.R., et al. (2023). Clinical applicability of the Polygenic Risk Score for breast cancer risk prediction in familial cases. J. Med. Genet. *4*, 327–336. https://doi.org/10.1136/jmg-2022-108502.
17. Wu, P., Gifford, A., Meng, X., Li, X., Campbell, H., Varley, T., Zhao, J., Carroll, R., Bastarache, L., Denny, J.C., et al. (2019). Mapping ICD-10 and ICD-10-CM Codes to Phecodes: Workflow Development And Initial Evaluation. JMIR Med. Inform. *7*, e14325. https://doi.org/10.2196/14325.
18. Johnson, R., Ding, Y., Venkateswaran, V., Bhattacharya, A., Boulier, K., Chiu, A., Knyazev, S., Schwarz, T., Freund, M., Zhan, L., et al. (2022). Leveraging genomic diversity for discovery in an electronic health record linked biobank: the UCLA ATLAS Community Health Initiative. Genome Med. *14*, 104. https://doi.org/10.1186/s13073-022-01106-x.
19. Lambert, S.A., Gil, L., Jupp, S., Ritchie, S.C., Xu, Y., Buniello, A., McMahon, A., Abraham, G., Chapman, M., Parkinson, H., et al. (2021). The Polygenic Score Catalog as an open database for reproducibility and systematic evaluation. Nat. Genet. *53*, 420–425. https://doi.org/10.1038/s41588-021-00783-5.

HGGA, Volume *5*

Supplemental information

Generalizability of PGS_{313} for breast cancer risk in a Los Angeles biobank

Helen Shang, Yi Ding, Vidhya Venkateswaran, Kristin Boulier, Nikhita Kathuria-Prakash, Parisa Boodaghi Malidarreh, Jacob M. Luber, and Bogdan Pasaniuc

Supplemental List

Figure S1: Kaplan Meier Curve for Overall Survival in European Patients with or without Chemotherapy Treatment

Figure S2: Kaplan Meier Curve for Overall Survival in European Patients above or below the 70th Percentile of PGS_{313}

Figure S3: Multivariable Cox Proportional Hazards Model on OS in European Patients

Figure S4: Genetic Admixture across GIAs

Table S1: Medications used for identifying HR+ breast cancer cases

Table S2: Medications used for identifying HER2+ breast cancer cases

Table S3: Association between PGS and Breast Cancer Risk in GIAs

Table S4: AUCs by GIA

Table S5: Downsampling Experiment

Supplemental Material and Methods

Supplemental References

Figure S1: Kaplan Meier Curve for Overall Survival in European Patients with or without Chemotherapy Treatment

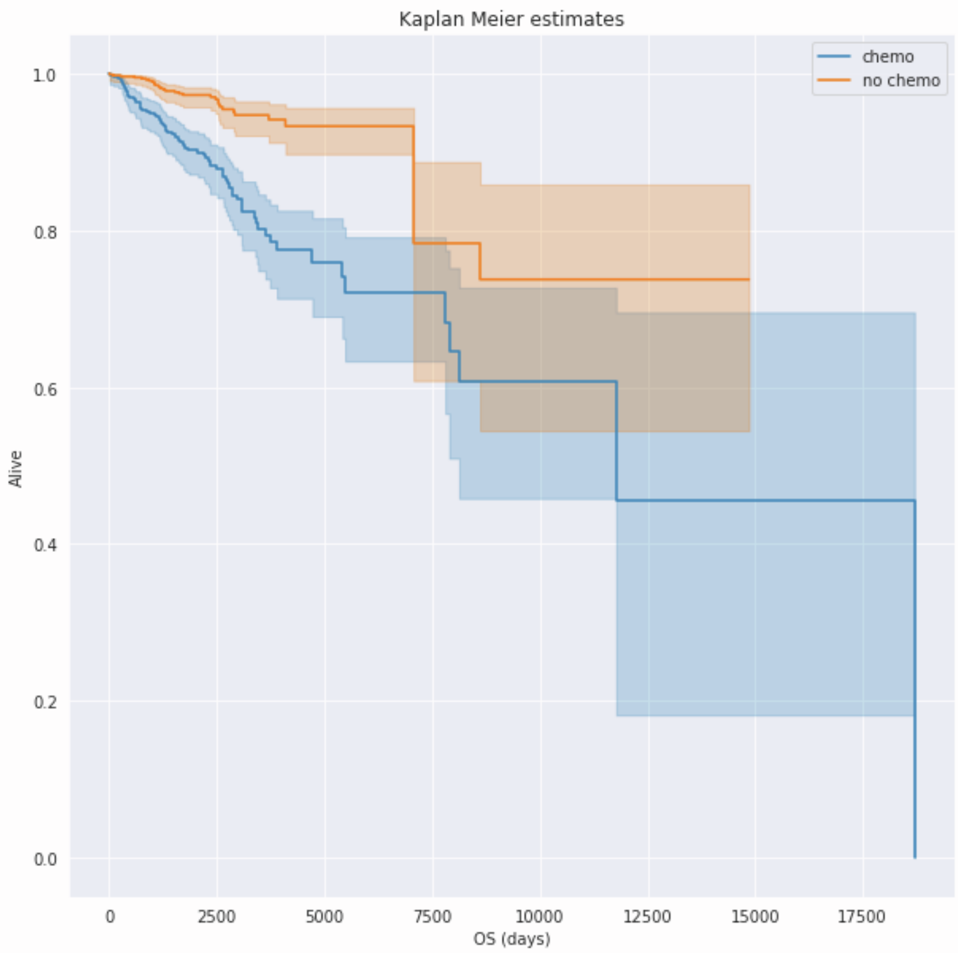

There were 503 patients who had received chemotherapy and 767 without chemotherapy in our European cohort. By Kaplan Meier survival analysis, we confirmed our approximate OS values were appropriately shorter in all patients who had received chemotherapies for breast cancer relative to those who had not (p value < .005). This is expected as patients who receive chemotherapy often have more aggressive or metastatic disease, resulting in shorter survival.

Figure S2: Kaplan Meier Curve for Overall Survival in European Patients above or below the 70th Percentile of PGS_{313}

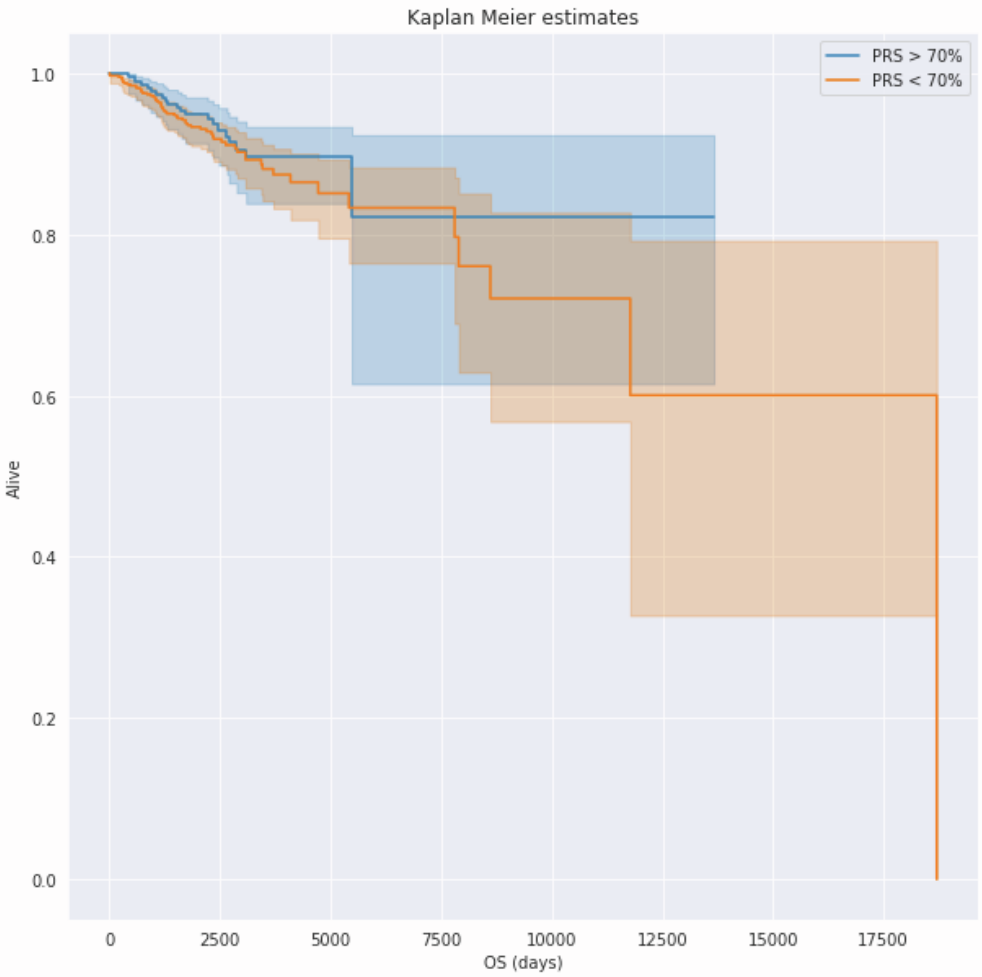

To evaluate the impact of PGS_{313} on OS, we compared survival times by Kaplan Meier analysis for European patients above (N=280) and below (N=651) the 70th percentile of the PGS. We found no difference in survival time between the two groups by log-rank test (p-value= 0.38). There was also no difference in survival time when comparing above and below the 50th percentile as well as the top 90th and lowest 10th percentiles.

Figure S3: Multivariable Cox Proportional Hazards Model on OS in European Patients

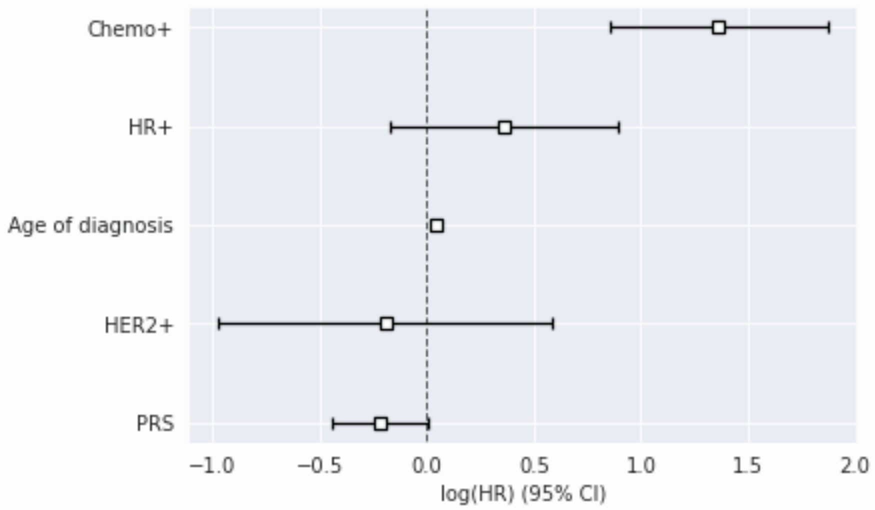

For European patients, we initially found by Cox Proportional Hazards that the normalized PGS_{313} was inversely predictive of OS, suggesting that a lower PGS_{313} score translates to longer survival time (HR, 0.80; 95 CI, 0.64-0.99, p-value = 0.04). However, when adjusted for other variables such as whether or not a patient had received chemotherapy, cancer subtype (HR+ and/or HER2+), and age of diagnosis, the normalized PGS_{313} score is no longer predictive of OS (p–value = 0.06).

Figure S4: Genetic Admixture across GIAs

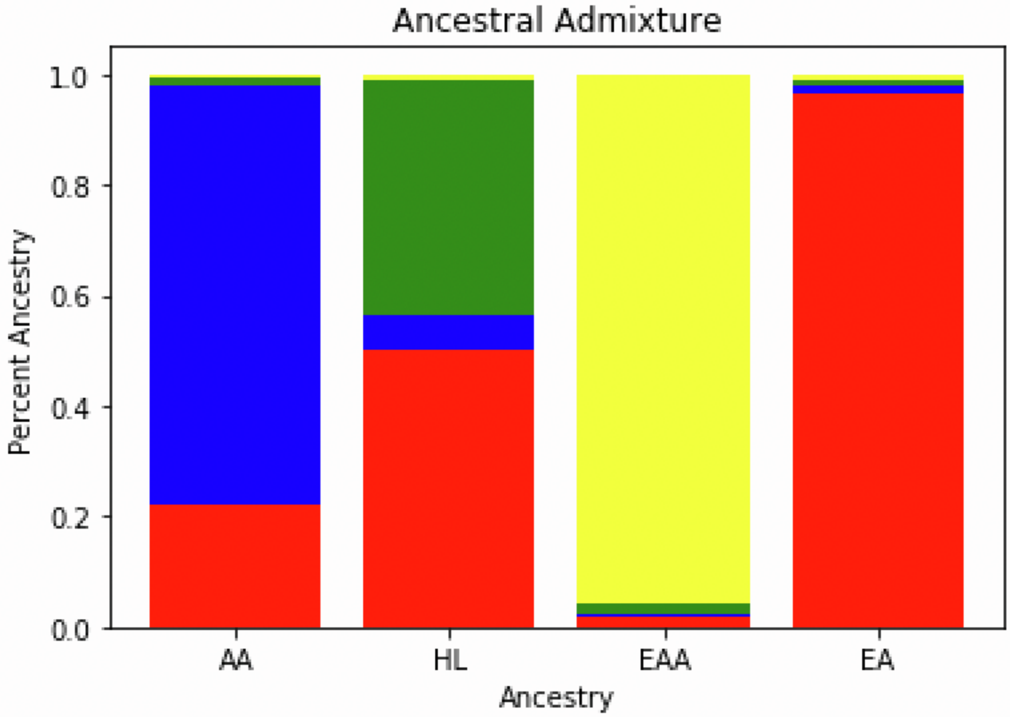

We found that genetic admixture was present but in varying degrees. The EAA population had the least overlap with the EA population, whereas the HL population had the most overlap with the EA population. These results suggest that genetic admixture may explain the overlapping performance of the PGS on our HL and EA cohorts.

Table S1: Medications used for identifying HR+ breast cancer

Name	Medication Class
Exemestane	Hormone therapy
Anastrazole	Hormone therapy
Letrozole	Hormone therapy
Tamoxifen	Hormone therapy
Raloxifine	Hormone therapy
Fulvestrant	Hormone therapy
Palbociclib	CDK 4/6 inhibitor
Ribociclib	CDK 4/6 inhibitor
Abemaciclib	CDK 4/6 inhibitor
Everolimus	mTOR inhibitor
Alpelisib	PI3K inhibitor

Table S2: Medications used for identifying HER2+ breast cancer

Name	Medication Class
Trastuzumab	HER2 monoclonal antibody
Pertuzumab	HER2 monoclonal antibody
Neratinib	Tyrosine Kinase Inhibitors
Lapatinib	Tyrosine Kinase Inhibitors
Ruxolitinib	Tyrosine Kinase Inhibitors
Osimertinib	Tyrosine Kinase Inhibitors
Tucatinib	Tyrosine Kinase Inhibitors
Niraparib	Tyrosine Kinase Inhibitors
Dasatinib	Tyrosine Kinase Inhibitors

Table S3: Association between PGS and Breast Cancer Risk in Genetically Inferred Ancestries (GIA)

GIA	OR	Lower CI	Upper CI
AA	1.31	1.05	1.64
EA	1.52	1.23	1.72
EAA	1.46	1.43	1.61
HL	1.51	1.31	1.75

Table S4: AUCs by Genetically-Inferred Ancestry

GIA	AUC	Lower CI	Upper CI
AA	0.613	0.567	0.659
EA	0.650	0.621	0.680
EAA	0.620	0.581	0.658
HL	0.688	0.676	0.700

We found overlapping AUCs for the HL and EA cohorts, as determined by their overlapping 95% confidence intervals. The AUCs for the AA and EAA populations were lower as determined by their non-overlapping 95% confidence intervals, relative to the EA and HL population.

Table S5: Downsampling Experiment

GIA	Batched OR	Batched Lower CI	Batched Higher CI	Raw OR
AA	1.40	0.73	2.07	1.31
EA	1.64	0.74	2.38	1.51
EAA	1.29	0.65	1.93	1.46
HL	1.69	0.97	2.66	1.52

To evaluate if differences in ORs were due to sample size imbalance across the GIAs, we conducted an ensemble downsampling of all groups in 500 batches. The averaged OR and 95% CI (see Batched OR and Batched CI) overlapped with all of the Raw ORs for each GIA, as determined by the overlapping 95% confidence intervals, suggesting that differences in OR observed in our AA cohort, relative to our EA cohort, are less likely due to differences in sample size. However, for the EA cohort, the batched 95% CI was wider than that observed for the raw data, suggesting that a component of the larger 95% CI spread for the AA cohort may be due to sample size, as expected.

Supplemental Material and Methods

Kaplan Meier Curves

We approximated Overall Survival (OS) in days by subtracting the present day or date of death from date of diagnosis amongst patients who were still living or dead, respectively, in the EHR. To confirm that our estimates of OS were reliable, we then compared survival time amongst these two groups by Kaplan Meier analysis to demonstrate that patients who had undergone chemotherapy had shorter survival times, as expected. Only European patients were chosen for this analysis due to fewer patients amongst other GIAs that had received chemotherapy. Kaplan Meier analysis was also used to evaluate survival time amongst European patients at the top 70th percentile of the PGS_{313} versus those below this threshold. The 70th percentile was chosen as starting at this threshold, the OR was noted to be statistically greater than 1 for the EA cohort (Figure 2).

Cox Proportional Hazards Model

Cox Proportional Hazards was used to model PGS_{313}'s effect in combination with other co-variates on OS. This would also allow us to confirm if there might be any cofounders of the $PGS_{313.}$ Due to limited cohort numbers, we chose to only model the European population, which had sufficient numbers of patients who had undergone chemotherapy and were HER2+. For our Cox Proportional Hazards model, we included whether or not a patient had received chemo, HER2 status, normalized PGS_{313} and our estimated age

at diagnosis based on when an ICD code related to breast cancer first appeared in the EHR.

Subtyping

We first queried the top 50 most commonly prescribed cancer-related medications within the electronic medical record for our cohort in the EHR. These were manually reviewed and grouped based on class and subtype relevance. Supplemental Table 1 shows our grouped list of HR+ relevant medications and Supplemental Table 2 shows our grouped list of HER2+ relevant medications. Patients were subtyped as HR+ and/or HER2+ if they had been ordered at least one relevant medication from each list in the EHR.

Genetic Admixture

Please refer to our prior publication regarding our methodology for our genetic admixture analysis using k=4 subpopulations, representing the number of genetically inferred ancestries (GIAs) in our study[1].

Association between PGS and Breast Cancer Risk in Genetically Inferred Ancestries (GIA)

Univariate logistic regression was used to evaluate the association between PGS_{313} and observed rates of all breast cancers in American females of African (AA), European (EA), East Asian American (EAA), and Hispanic (HL) genetically inferred ancestry. As with prior studies testing the generalizability of PGS_{313}, we normalized the raw PGS

score of non-European GIAs based on the average and standard deviation of European samples. In comparison, Mavaddat et al. reported an OR of 1.61 (95 CI 1.57-1.65), which overlaps with all GIAs.

AUCs by Genetically-Inferred Ancestry

Logistic regression was performed to predict the labeling of breast cancer status using the normalized PGS, Age at diagnosis, and PCs 1-9 as co-variates, as consistent with Mavaddat et al. AUCs were calculated for each of the four different GIAs separately.

Downsampling experiment

To confirm that differences in ORs were not due to sample size imbalance across the GIAs, we conducted an ensemble downsampling of all groups such that all were downsampled into 500 batches of 124 cases and 124 controls each, which were randomly selected, as this was the number of cases in the AA cohort, which had the fewest number of cases across all GIAs. We then calculated the average OR and 95% CI across all 500 experiments (see Batched OR and Batched CI) and compared this with the Raw ORs for each GIA.

Supplemental References

1. Johnson R, Ding Y, Venkateswaran V, et al. (2022). Leveraging genomic diversity for discovery in an electronic health record linked biobank: the UCLA ATLAS Community Health Initiative. Genome Med *1*, 104. 10.1186/s13073-022-01106-x.
